# Rapid growth of antimicrobial resistance: the role of agriculture in the problem and the solutions

**DOI:** 10.1007/s00253-022-12193-6

**Published:** 2022-10-05

**Authors:** Dragana Stanley, Romeo Batacan, Yadav Sharma Bajagai

**Affiliations:** Institute for Future Farming Systems, Central Queensland University, Rockhampton, QLD 4702 Australia

**Keywords:** Antibiotic, Resistance, Agriculture, Hospital, Manure

## Abstract

**Abstract:**

The control of infectious diseases has always been a top medical priority. For years during the so-called antibiotic era, we enjoyed prolonged life expectancy and the benefits of superior pathogen control. The devastating failure of the medical system, agriculture and pharmaceutical companies and the general population to appreciate and safeguard these benefits is now leading us into a grim post-antibiotic era. Antimicrobial resistance (AMR) refers to microorganisms becoming resistant to antibiotics that were designed and expected to kill them. Prior to the COVID-19 pandemic, AMR was recognised by the World Health Organization as the central priority area with growing public awareness of the threat AMR now presents. The Review on Antimicrobial Resistance, a project commissioned by the UK government, predicted that the death toll of AMR could be one person every 3 seconds, amounting to 10 million deaths per year by 2050. This review aims to raise awareness of the evergrowing extensiveness of antimicrobial resistance and identify major sources of this adversity, focusing on agriculture’s role in this problem and its solutions.

**Keypoints:**

• *Widespread development of antibiotic resistance is a major global health risk.*

• *Antibiotic resistance is abundant in agricultural produce, soil, food, water, air and probiotics.*

• *New approaches are being developed to control and reduce antimicrobial resistance.*

## Introduction

The UK review of antimicrobial resistance (AMR) presented now outdated 2014 data on AMR consequences for human life. They estimated that 700,000 people die every year from AMR. This prediction was recently updated with the data from 2019 presenting that the deaths of 4.95 million people were related to drug-resistant bacterial infections and 1.27 million deaths were directly attributable to AMR (Antimicrobial Resistance 2022; Lancet 2022); data was based on 471 million individual records. The estimated death rate was highest in Africa and lowest in Australasia.

In India, a country with high antibiotic use and high AMR, approximately 60,000 newborn babies die each year from antibiotic-resistant neonatal infections (Chaurasia et al. 2019; Laxminarayan et al. 2013). The profile of the most devastating pathogens involved in neonatal sepsis in South Asia points to the dominance of Gram-negative infections; *Klebsiella* sp., *Escherichia coli* and *Acinetobacter* sp. leading the list, with *Staphylococcus aureus* as the most lethal Gram-positive (Chaurasia et al. 2019). With soaring and out of control global expansion of AMR, it is not surprising that most of these pathogens are multidrug resistant (MDR) and that the victims are very young children and elderly, hinting at the dire predictions that AMR could return the world to middle ages, where high childbirth and neonatal mortality could become a reality again.

**Fig. 1:**
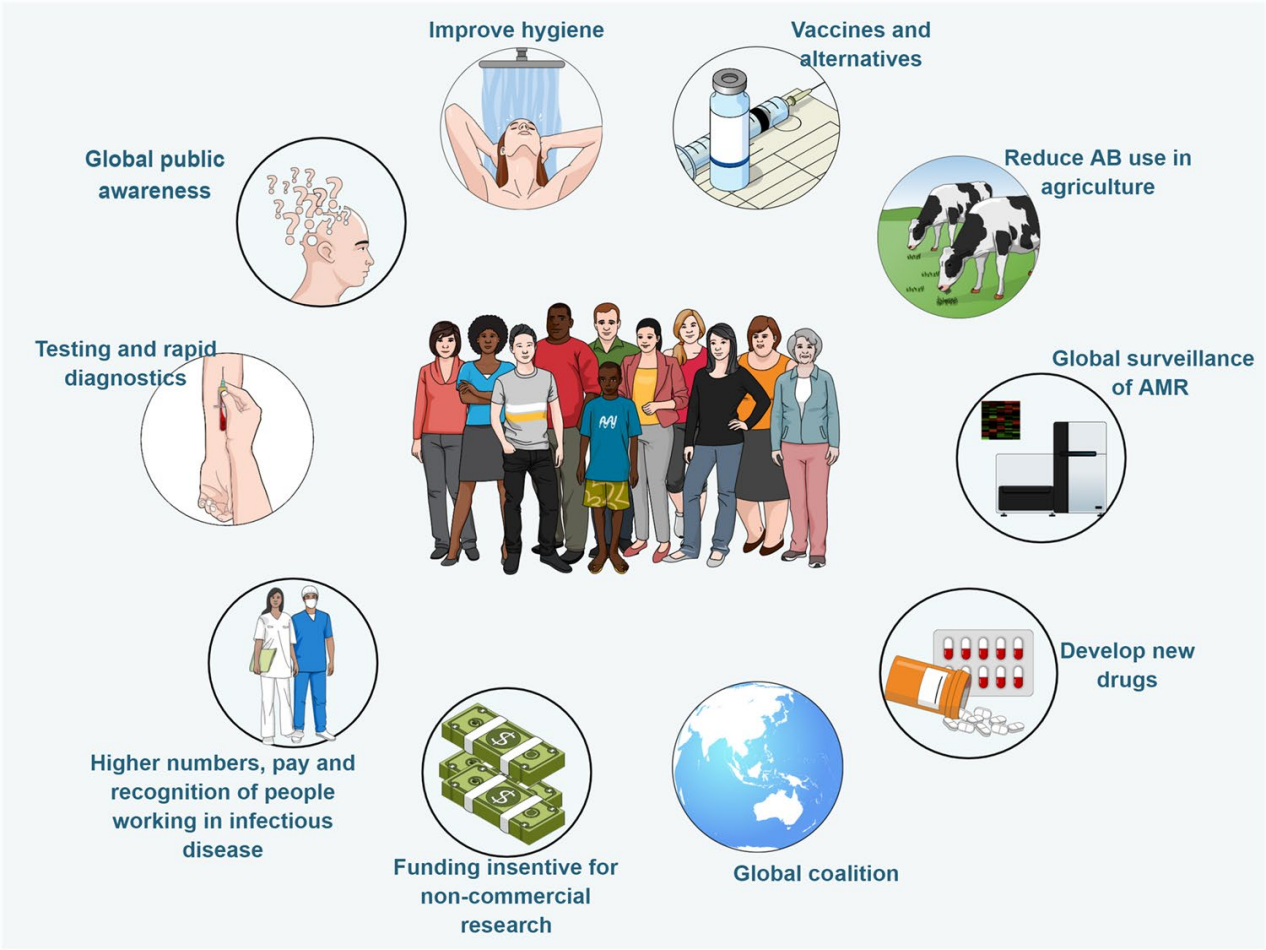
Recommendations from The Review on Antimicrobial Resistance (O’Neill 2016). The figure was created using Biorender

The AMR problem is more prominent in low and middle-income countries but by no means restricted to them. There were more than two million AMR infections in the USA in 2016, costing the USA health system 20 billion USD (O’Neill 2016). The UK AMR committee recommended a strong response to AMR (Fig. 1); however, other than agriculture, which was required in many countries to respond to AMR threat and remove antibiotics from production altogether, other directives are lagging far behind on implementation. Some of these directives are very basic, like growing public awareness, improving diagnostics and hygiene worldwide, or continuing to work on antibiotic alternatives, these are suggestions that do not seem like an impossible or unrealistic task, and they are slowly moving forward. Other more complex and funding/investment dependent recommendations like the global coalition, increased funding for research on controlling AMR, better AMR diagnostics and speeding up the development of new antibiotics and vaccines are not progressing as expected, and the lack of progress suggests that these directives were not taken seriously enough. Getting all countries in the world to unite and work together on one task, even if it is survival related, is a mission impossible in a divided world, and pharmaceutical companies are no longer seeing the development of new antibiotics as financially viable.

Many governments have antimicrobial stewardship plans, with some countries moving from words to hard-hitting actions, for example, the ban on nontherapeutic antibiotic use in livestock (Levy 2014) that started in Scandinavia and spread to other countries of the European Union. India, one of the countries in the world known for very high antibiotic use, came up with India’s National Action Plan (NAP) (2017) with objectives comparable to those of O’Neill (2016), and the improvements are already visible (Ranjalkar and Chandy 2019). Although low and middle-income countries are also taking individual approaches to AMR control, there is no global coordination, and as our review will show below, AMR knows no geographic boundaries, and colossal efforts by some countries are undermined by the mild response of others.

Progress in developing diagnostic tools is very slow, and rapid molecular genomics methods are not optimised and developed for mass distribution. Microbial culture followed by susceptibility testing seems to be a widely used solution, and the time delay in identifying the correct treatment can mean the difference between life and death. Global surveillance of AMR is manifested by a range of publications cataloguing AMR genes in different clinical pathogens using hospital samples and data on AMR presence in all aspects of the environment.

The majority of work on alternatives to antibiotics came from the livestock industry (pig, chicken and cattle) because the ban on antibiotic use for growth-promoting purposes resulted in an increase in disease outbreaks since the antibiotics also kept pathogens in check while improving growth performance measures. This dire need induced the industry to invest in research on alternatives to antibiotics, and the market is now flooding with antimicrobial products that have no overlap with antibiotics. Among the most successful and widely used are phytogens, plant antimicrobial products whose antibacterial properties can successfully provide growth-promoting and pathogen controlling effects (Abdelli et al. 2021; Shehata et al. 2022).

In addition to searching for natural alternatives to antibiotics that would not result in AMR cross-resistance, the industry introduced significant changes toward improving animal health, welfare and on-farm biosecurity. On the other hand, the customer-driven shift toward free and open-range production systems aimed at improving animal welfare is backfiring via increased disease outbreaks caused by direct contact of animals with wildlife, insects, soil bacteria, rodents, wild birds, predators and other animals (Phung et al. 2019). The industry also offers a range of new products capable of disturbing biofilms in sheds and equipment or controlling pathogen growth in the litter. However, although it is reasonable to expect that these products would assist in the control of AMR, evidence of this is expected to develop in the future.

Developing new antibiotics seems to be slowly moving from the long break in the 1990s that, not surprisingly, coincides with the beginning of the rise of AMR. Despite having a few new antibiotics in the clinical trial stage, and although they are efficient against some of the major MDR pathogens, they are not based on a novel mode of action and the AMR against them is likely to develop fast (Bettiol and Harbarth 2015). Developing new classes of antibiotics with a novel mode of action would be the only longer-term solution with likely more use time before the AMR genes appear; however, this is now considered a low or no return investment due to the high risk and cost of product development. With antibiotics being steadily turned ineffective by MDR bacteria, the alternative of focusing on the immune response using vaccines and therapeutic monoclonal antibodies is starting to look more attractive.

## Major contributors to AMR rise

Despite some effort from developed countries to monitor and reduce AMR, the lack of global effort and international coordination is concerning. The current situation could be defined as the saturation of the human environment, including water, soil, food and air, with high levels of AMR. Major contributors include human and hospital waste and agriculture.

### Human and hospital waste

The sewage microbial community comprises mainly human intestinal bacteria and some bacteria that grow on the sewage equipment and system. Antibiotics are developed to be resilient to metabolic degradation and active during biological transit time. It is estimated that 50–80% of antibiotics is excreted in urine and 4–30% in faecal material (Riaz et al. 2020). This indicates that all unmetabolised antibiotics used by the human population, at home and in hospitals, end up in the sewage system. The sewage is saturated with antimicrobial-resistant bacteria (ARB) and their antimicrobial-resistant genes (ARGs). The troubling issue is the abundance of mobile genetic elements (MGEs), such as plasmids, which serve as vectors transferring ARGs between bacterial strains in the sewage environment. Sewage is the breeding ground for new ARBs via ARG transfer.

Unfortunately, wastewater treatment plants (WWTPs) were not designed to remove or reduce AMR contamination (Hiller et al. 2019). On the contrary, some MDR species of *Escherichia*, *Shigella* and *Klebsiella* increase two-fold from raw to treated water. Similarly, total MDR *Enterobacteriaceae* surged from 5.5 to 14.1% in the treated wastewater. Species like methicillin-resistant *Staphylococcus aureus* (MRSA) are abundant in raw and treated sewage (Krzemiński et al. 2020). Hospitals often have their own waste treatment facility. However, that is reserved for medical waste rather than the general waste from toilets, kitchens and laundry. There is a whole new level of AMR enrichment in a hospital environment. Here, it is important to note that all hospital waste eventually ends up in sewage and WWTP.

### AMR from the agriculture

Agriculture is commonly acknowledged as one of the principal contributors to AMR pollution. With Denmark and other countries of the European Union banning the use of antibiotics in livestock (Levy 2014), the stunned livestock industry finally recognised the urgent need for antibiotic alternatives. Continual use of antibiotics in animal feed controls pathogens and improves performance, but it also alters microbial community depending on the product used (Crisol-Martinez et al. 2017). This can be detrimental in poultry since the industrialised hatching practices involve separating eggs from the mother hen, disinfecting them and hatching in clean and bacteria-deprived environment, resulting in unnatural microbial communities that can only further suffer from continual supplementation of antibiotics in the feed (Stanley et al. 2013). The industry invested in alternative and natural antimicrobial products capable of controlling pathogens, such as biochar (Willson et al. 2019), zeolite (Prasai et al. 2017) or plant-derived complex phytogenic products (Bajagai et al. 2020; Flees et al. 2021; Greene et al. 2021; Wang et al. 2021). Recently, using a mixed phytogenic alternative product, RNAseq analysis showed that herb-based antibiotic alternative triggers ileum gene expression response significantly matching the response to doxycycline and geldanamycin (Bajagai et al. 2022), indicating a broad spectrum of the antibiotic alternative products. The main products of the livestock industry, such as meat, milk or eggs, are meant for human consumption; however, byproducts such as manure play a significant role in other areas of agriculture.

Three major manure types are used in agriculture: bovine, pig and poultry. Although animal manures enrich the soil with essential and rare plant nutrients, they also contain biological impurities that include bacteria, fungi, helminths, parasites and other intestinal and environmental biological contaminants. Antibiotic use in agriculture is a major issue in developing countries (Hosain et al. 2021). Residual concentration of antibiotics in manure may not be very high and is reported commonly as ~ 1–10 mg/kg (Krzemiński et al. 2020); it was estimated that in the last 50 years, more than 1 MT of antibiotics entered the soil via manure (Allen et al. 2010).

The animal manure contains both urinary and faecal excrements, and both are used to excrete unmetabolised antibiotics. In addition to adding antibiotics to the soil via manure, we also add ARB and ARGs. The survival time of pathogens in the soil is up to 10 years, and on plant surfaces, up to 1 year (Krzemiński et al. 2020). Defra project by UK Centre for Ecology & Hydrology (Nicholson et al. 2016) estimated that approximately 96 million tonnes of farm manure are used annually in the UK. With this volume of manure applied worldwide, antibiotic accumulation in the soil is inevitable.

**Fig. 2:**
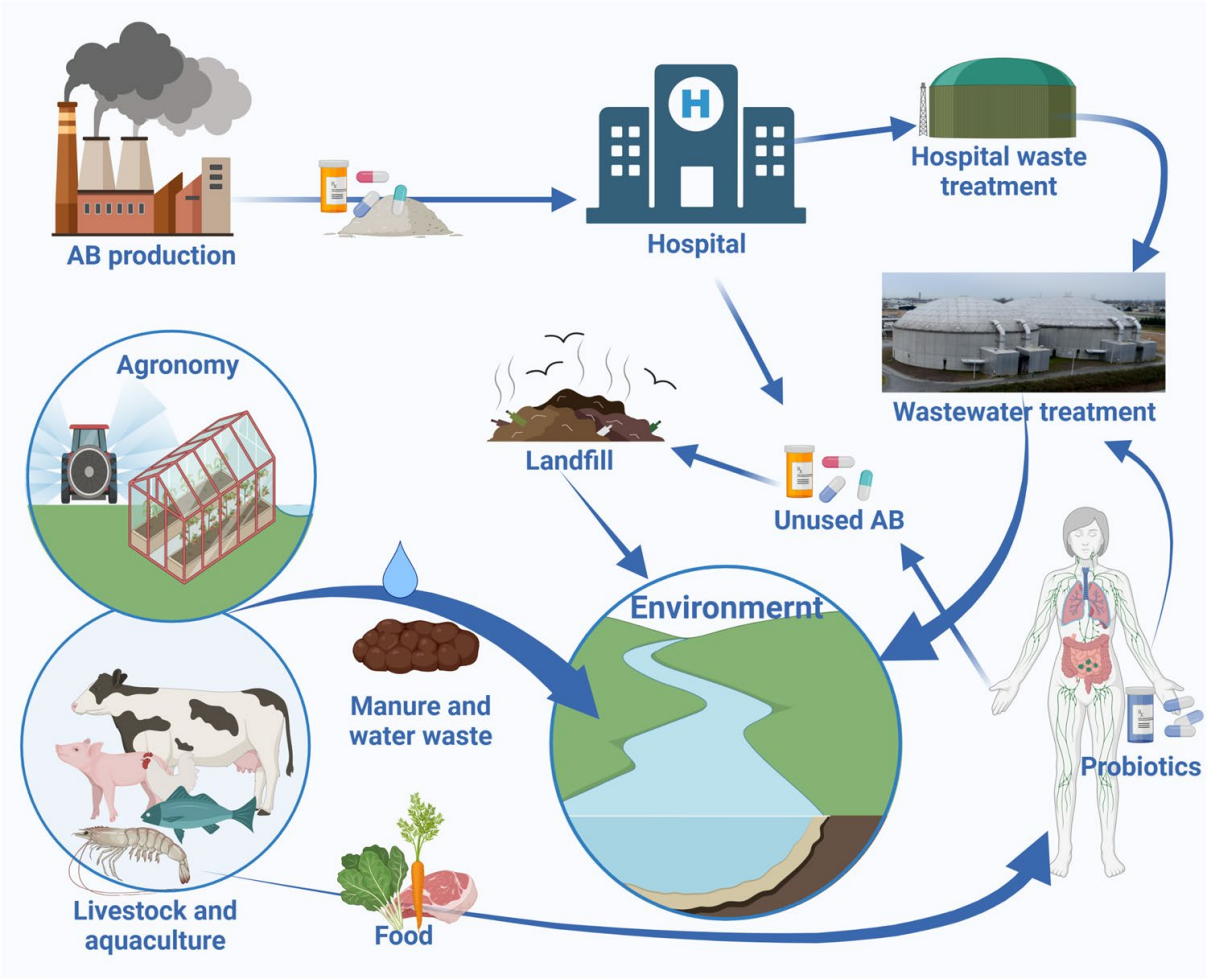
Vicious cycle of AMR spread and expansion in the environment. The figure was created using Biorender

Although manure is a major source of antibiotics, ARG and ARB in the soil, in the USA and many other countries, all manure is considered as an organic product and it can be used on crops grown organically, thus introducing residual excreted antibiotics into organic plants and allowing AMB to colonise plants and ARGs exchange with native farm soil microbiota. As a result, many organic plants carry an abundance of AMR (Roberts 2019). Manure and faecal effluent processing are highly variable, not only on the global scale but also from one farm to another in the same country. Ultimately, all of these biological contaminants end up in food and waterways (Fig. 2).

There is another dimension of the presence of AMR in pig and chicken sheds — farm employees’ welfare and safety. A good example is the UK use of vancomycin-similar drug avoparcin in poultry which resulted in the development of vancomycin-resistant *Enterococcus faecium* (VRE) in chicken. VRE was then found in processed meat and farmers and abattoir staff slaughtering the VRE + animals, some of whom had to be hospitalised with the VRE infection (Cetinkaya et al. 2000; van den Bogaard et al. 2002). Additionally, van den Bogaard et al. (2002) reported that AMR for nearly all antibiotics was higher in broiler farmers than in egg farmers and slaughterhouse workers. This is in agreement with the higher use of antibiotics in broilers. Moreover, it was reported by multiple investigators that in cattle and swine, faecal AMR decreases as food animals age, but this was not observed in poultry with short production life (Gaire et al. 2020).

Despite much attention to livestock and manure, one of the biggest AMR offenders is aquaculture (Krzemiński et al. 2020). Aquaculture is the fastest-growing food-producing area in the world and is considered by many the future of the food industry. Integrated aquaculture that involves feeding fish livestock manure is a traditional practice of the smaller Asian farms. Tetracyclines were extensively used in aquaculture, and aquaculture is mainly blamed for the broad tet-genes’ distribution in the world. In 2017, 59 tetracycline resistance genes were identified even in the world’s most pristine remote environments like Antarctica and the Arctic (Roberts 2019). It was estimated that 70% of an enormous amount of antibiotics administered in aquaculture end up in waterways. In addition to using and releasing massive amounts of antibiotics into waterways, the aquaculture AMR bio-waste contaminates adjacent soils and waterways’ sediments where it is actively concentrated.

With the rise in seafood consumption, seafood-borne bacterial diseases are becoming more severe health hazards worldwide, and due to antibiotic use in production, it resulted in numerous AMR pathogens outbreaks in the USA (Elbashir et al. 2018). However, there is a considerable discrepancy in antibiotic usage in different countries. For example, antibiotics are used in a wide range — from 1 g/t in Norway to 700 g/t in Vietnam (Defoirdt et al. 2011). Strict government-controlled surveying and transparent antibiotic use reporting in Scandinavian countries, with high use of alternatives to antibiotic use, can be credited for this outstanding practice in Norway (Defoirdt et al. 2011; Krzemiński et al. 2020; Narbonne et al. 2021). Asian countries yield two-thirds of global food fish production, yet concerning levels of resistance to clinically critical antibiotics and high levels of foodborne zoonotic pathogens are evident (Schar et al. 2021), and it resulted in accumulated resistance along with Asia’s major river systems, especially in China and India (Schar et al. 2021). Furthermore, AMR, ARB and ARGs are regularly present in drinking water (Roberts 2019).

### AMR in the soil

Soil represents one of the richest microbiota systems, abundant with bacterial and fungal communities, and it is the origin of many clinical antibiotics (Afzaal et al. 2020). Similar to waterways, the soil acts as a major sink that accepts the AMR from major contaminants like hospital waste, wastewater treatment, aquaculture and manure. Although some antibiotics can chemically bond with the soil and persist, soil factors such as soil texture and AMR gene stability play a role in AMR persistence in the soil (Macedo et al. 2020). In addition to antibiotics, many metals in the soil that generate toxic metal resistant bacteria and toxic metal resistant genes show potent cross- and co-resistance to antibiotics and can result in AMR gene emergence without any exposure to antibiotics (Yazdankhah et al. 2018). This is further complicated with both manure and inorganic fertilisers.

### AMR in the food

AMR pathogens in human food present a major risk to public health. Food is contaminated by the presence of antibiotics and ARB on food due to the use of antibiotics in production and cross-contamination processing (Verraes et al. 2013). This is of special concern with seafood (Elbashir et al. 2018). ARB and ARG have been identified in tap water and feed, including milk, eggs, meat and vegetables, all of them as processed and unprocessed foods (Roberts 2019); thus, the food chain is one of the principal means of AMR transmission. Although often present in a very low amount on meat, ARBs can often amplify to high numbers due to poor meat handling, storage and hygiene practices (Plaza-Rodriguez et al. 2021).

While ARB and AMR in the meat are a consequence of the contamination in slaughterhouses and processing, vegetables are known to actively uptake antibiotics and AMR genes from the soil or irrigation water (Azanu et al. 2016), especially if the manure is used for fertilising (Tasho and Cho 2016). Wang et al. (2015) and many others reviewed in Tasho and Cho (2016) identified highly abundant ARGs on vegetables growing on soil with more than 3 years of manure usage.

Although very little attention is given to wild animals, the levels of AMR infiltration into their habitat through water, soil, and plants suggest that they could not remain unaffected. Even in wild animals that have minimal contact with humans and live in generally considered pristine environments, ARB, like highly multiresistant MRSA, were detected in dear, boar, wolfs, foxes, pigeons, pheasants and birds of prey (reviewed in Silva et al. 2020).

Fruit and vegetables are declared safe for human use if they have antibiotic residue under the selected maximum threshold. Similarly, fish and seafood heavily treated with antibiotics can be used after being housed in antibiotic-free water for some time, referred to as the withdrawal period (Bhattacharjee 2016). Unfortunately, the antibiotic residue is a much smaller problem than ARB and ARGs in food. Additionally, washing fruit and vegetables in drinking tap water will not help since many researchers detected AMR in tap water (Roberts 2019).

On the other hand, no food is ever sterile, and there are three major types of microbial contamination: resident food microbiota, pathogens and spoilage bacteria. Throughout history, people relied on residential food bacteria to start the process of natural fermentation. Whole grains carry a range of probiotic lactic acid bacteria (Carrizo et al. 2016; Wu et al. 2018) with a range of *Lactobacillus Pediococcus*, *Leuconostoc* and *Enterococcus* species capable of performing sourdough fermentation. Bacterial and fungal endophytes are known as plant probiotics and are extensively documented in grains (Ahlawat et al. 2021; Makar et al. 2021) and legumes (Ruiz Mostacero et al. 2021). Recently microbiota of poultry feed raw ingredients such as meat and bone meal, wheat, corn, canola, barley, soybean, millrun, sorghum, poultry oil, oats, limestone and bloodmeal was described using the 16S methodology (Haberecht et al. 2020).

Likewise, animals’ microbiota is not limited to the intestinal community, and the use of advanced sequencing methodologies challenged the concept of sterile organs, demonstrating that stressful events can trigger massive leaky gut events capable of colonising and infecting the host’s organs (Amar et al. 2011; Stanley et al. 2016). *Proteobacteria* and *Firmicutes* are the main members of human blood microbiota, with *Proteobacteria* present in the blood of more than 80% of healthy human subjects (Paisse et al. 2016). The involvement of tissue bacteria has been investigated in the onset of diseases such as diabetes (Amar et al. 2011) and stroke (Stanley et al. 2016). Furthermore, food processing often adds pathogenic and food spoilage microorganisms and their growth is highly dependent on the storage and packaging conditions and meat residential microbiota structure (Patarata et al. 2020). Despite relatively recent knowledge on natural food microbiota, the bulk of research in this area is focused on foodborne pathogens, ARGs and AMR, without acknowledging the contribution of natural and beneficial microbiota in food products towards AMR load. It is well established that AMR is not exclusive to pathogens but that even probiotic species carry and disseminate AMR (Baumgardner et al. 2021). This could be highly overestimating the role and danger of AMR in the feed, and the actual risk of AMR in food should be further investigated, taking into account the origin of the AMR detected.

### AMR in the air

Although it may seem implausible, AMR and ARGs have been isolated from the air, especially in highly industrialised countries. Li and colleagues (2018) conducted a well-known study investigating air samples from 19 major cities worldwide and screening 30 ARGs. The ARGs were detected to vary by nearly 100-fold in different cities. The highest number of ARGs was detected in Bejing, and San Francisco had the highest ARG abundance. Antibiotic resistance genes conferring resistance to clinically critical antibiotics such as vancomycin were detected in air samples (Li et al. 2018), indicating that urban air represents a health risk associated with continual exposure to airborne ARGs. Li and colleagues (2018) also analysed bacterial communities in the air, their networks and contributions to ARGs. They found 50 genera in the urban air, including *Corynebacterium*, *Escherichia/Shigella*, *Streptococcus* and other potentially pathogenic genera.

The airborne AMR emission of 8 AMR-related genes from 163 layer farms near Beijing was quantified in Gao et al. (2022). The authors developed the atmospheric transport model with a gene degradation module for the assessment of the spatial and temporal distribution of poultry AMR while evaluating their regional exposure and sedimentation. Their findings demonstrate the deposition of AMR in residents’ upper respiratory tract, highlighting that the air is a much more hazardous AMR dissemination pathway than previously thought. The study by Xin et al. (2022) confirms the importance of the air as a dissemination pathway. They investigated pathways of 30 ARGs, four MGEs and four human pathogens from four animal species across 20 farms. Apart from the high concentration of these resistance markers in the air near the farms, they reported high inhaling exposure of farm workers. Unlike AMR in soil, the AMR in air and water are highly mobile, and diffusion, floods and rain play a role in spreading AMR, ARB and ARGs to the remote and “untouched” ecosystems.

### AMR in probiotics

Although often ignored, it is well established that most probiotics carry AMR and ARGs. This is well investigated and comprehensively reviewed (Gueimonde et al. 2013; Li et al. 2020). The probiotics carry ARGs because a complete AMR profile was initially not required for their registration. Authors universally agree that ARGs on mobile genetic elements, such as plasmids, constitute a significant problem because of the transfer of resistance to other potentially pathogenic species (Baumgardner et al. 2021; Zheng et al. 2017). Liu et al. (2009) confirmed the presence of antibiotic resistance, including clinically critical, in commercial probiotics, identifying vancomycin, rifampicin, streptomycin, bacitracin and erythromycin ARGs (Liu et al. 2009), and suggested new regulations to a re-evaluation of probiotic safety (Liu et al. 2009).

Probiotics are continually added into the intestinal community due to regular use in humans and continuous supplementation in livestock and poultry (Zheng et al. 2017), and the AMR accumulation effects are not yet investigated. Baumgardner and colleagues (2021) performed a comprehensive study to determine if transferable ARGs are present in 47 commercially available veterinary probiotics, including products marketed for cattle, dogs, camelids, cats, goats and horses. Ninety-seven percent of the 47 probiotics contained at least one mobile AMR gene, and 82% contained two or more. This study is highly relevant to poultry and other livestock, and it confirms the risk for transmission of these mobile AMR genes into the meat products, manure and environment.

Considering the scale of production and administration of probiotics, they also strongly contribute to AMR enrichment in manure and from manure to the soil, water and the environment. In a survey of fermented food starter cultures from 90 different sources in Switzerland, Kastner et al. (2006) confirmed AMR in the most common starter culture species. A recent study by Montassier et al. (2021) published in Nature Microbiology brings this story to another level. An administration of antibiotics increased the lower GI tract resistome. However, the addition of probiotics further exacerbated AMR and ARGs expansion in the gut mucosa by promoting the bloom of strains carrying vancomycin resistance genes different to the resistance genes carried by the probiotic strains. This can open an interesting new area of research on identifying supplements that expand or reduce resistome in human and animal intestines. This is a very tricky research area as it depends on contradicting questions — was AMR increased or reduced simply because the product affects specific taxa carrying that particular AMR gene, and would the AMR gene be reduced if another, product-unaffected taxa carried it? This means that precise in vitro experiments performed in continuous culture could be useful to establish mechanisms and causality before it was confirmed in vivo in complex and uncontrolled intestinal microbiota. Additionally, the faecal transplant can assist in reducing ARGs in the gut (Montassier et al. 2021). This could be quite variable depending on the donor AMR profile. The cycle of AMR spread on a global scale is presented in Fig. 2.

## Future directions

The estimated death toll from rising AMR is millions per year (O’Neill 2016), and medical practitioners are constantly reporting new all-resistant strains of pathogens while the AMR related annual mortality is on the steep rise (Antimicrobial Resistance 2022). There are numerous suggestions to improve monitoring, surveillance and diagnostics; however, efforts to reduce AMR load in the environment are urgently needed. In addition to alternative antimicrobial products and improved animal welfare and biosecurity combined with the strict control of antibiotic administration and other measures prosed by O’Neill and collaborators (O’Neill 2016), one of the emerging methods is bioremediation using living organisms that can remove or reduce ARG load in treated samples.

Bacteria and other microorganisms such as protozoa and yeast are the most promising and widely used for AMR and antibiotic bioremediation. However, this is based on complex microbial interactions and is not yet fully understood or optimised. To assist bioremediation, microorganisms must be able to survive and thrive under extreme conditions (e.g. oxidative pressure, nutrient depletion, osmotic stress) (reviewed in Apreja et al. 2021). It was reported that a combination of bacteria and microalgae could efficiently remove a range of antibiotics from the sludge, where the symbiotic interactions between bacteria and microalgae played a major role in the kinetics of antibiotic removal (da Silva Rodrigues et al. 2021). Although this area of research is relatively new, modern methodologies such as shotgun sequencing of antibiotic-resistant isolates revealed new enzymes capable of bioremediation (dos Santos et al. 2015). They opened an opportunity for the rapid development of bacterial libraries capable of removing antibiotics from the environment. *Bacillus* species, alone or in combination with other microorganisms, are among the most promising candidates (Al-Gheethi et al. 2019).

Phytoremediation is the removal of antibiotics using algae. *Cyanobacteria* are among the most interesting: they use light as an energy source and CO_2_ as a carbon source, also helping in carbon sequestration during the bioremediation process. Additionally, they are hardy, capable of growing under extreme conditions and capable of fixing up atmospheric nitrogen. Successful examples of algal bioremediation of antibiotics are well presented and reviewed in the literature (Grimes et al. 2019; Xiao et al. 2021; Zhou et al. 2021). It is, however, important to monitor the production of algal toxins before selecting the algae of choice for bioremediation.

Mycoremediation and phytoremediation are up-and-coming remediation techniques using fungi and plants to remove antibiotics from the environment. We previously discussed how plants accumulate antibiotics from the soil; although this is an issue for vegetable and other edible plant production, it is excellent in bioremediation.

One of the superior technologies capable of removing antibiotics, AMR and ARGs, is aerobic and anaerobic digestion using carefully selected microbial inoculums and optimised digestion conditions (reviewed in Waseem et al. 2020). The major advantages of using digesters are the reduction of greenhouse gas emissions and the production of renewable energy. If properly optimised, they can remove AMR and ARGs by degrading the DNA. This suggests the treatment of manure with digesters which can often be coupled with pelletising machines resulting in safe manure for the environment and farmers (Waseem et al. 2020).

## Concluding remarks

There is no doubt that AMR is one of the major global health issues. AMR is a natural phenomenon that existed for as long as microorganisms. The preservation of intestinal content in naturally and deliberately mummified human remains confirmed the presence of resistance genes to most known antibiotics, including those most recently discovered (reviewed in Santiago-Rodriguez et al. 2017). This notion that AMR was always there became an excuse for many to ignore the current global AMR pollution putting aside the fact that never in history have we had millions of tonnes of antibiotics and AMR pumped into the environment. Positive actions by the agriculture industry in removing antibiotics from the feed and field and replacing them with natural antimicrobial alternatives, improving the health and welfare of the animals and upgrading production systems towards better pathogen and AMR control, combined with continually reducing antibiotic administration to humans, animals and plants are promising trends. Actions taken by several European governments to provide transparency on the use of antibiotics that allows the consumer to select more sustainable product are exceptional. However, these actions are strongly variable on the global scale, and ignoring the AMR issue in any part of the world may impact the global dissemination of AMR.
